# High Resolution and Fast Processing of Spectral Reconstruction in Fourier Transform Imaging Spectroscopy

**DOI:** 10.3390/s18124159

**Published:** 2018-11-27

**Authors:** Weikang Zhang, Desheng Wen, Zongxi Song, Xin Wei, Gang Liu, Zhixin Li

**Affiliations:** 1 Xi’an Institute of Optics and Precision Mechanics, Chinese Academy of Sciences, Xi’an 710119, China; ven@opt.ac.cn (D.W.); songxi@opt.ac.cn (Z.S.); weixin@opt.cn (X.W.); liugang@opt.cn (G.L.); lizhixin2015@opt.cn (Z.L.); 2 University of Chinese Academy of Sciences, Beijing 100049, China

**Keywords:** spectrum reconstruction, Fourier transform imaging spectrometer, parallel computing, high performance, GPU

## Abstract

High-resolution spectrum estimation has continually attracted great attention in spectrum reconstruction based on Fourier transform imaging spectroscopy (FTIS). In this paper, a parallel solution for interference data processing using high-resolution spectrum estimation is proposed to reconstruct the spectrum in a fast high-resolution way. In batch processing, we use high-performance parallel-computing on the graphics processing unit (GPU) for higher efficiency and lower operation time. In addition, a parallel processing mechanism is designed for our parallel algorithm to obtain higher performance. At the same time, other solving algorithms for the modern spectrum estimation model are introduced for discussion and comparison. We compare traditional high-resolution solving algorithms running on the central processing unit (CPU) and the parallel algorithm on the GPU for processing the interferogram. The experimental results illustrate that runtime is reduced by about 70% using our parallel solution, and the GPU has a great advantage in processing large data and accelerating applications.

## 1. Introduction

In recent years, with the rapid development of imaging spectroscopy, the Fourier transform spectrometer has become one of the most important payloads in space exploration and component analysis [1,2,3,4,5,6]. This type of spectrometer, the Fourier transform imaging spectrometer or interference imaging spectrometer, has wide application in remote sensing, environmental monitoring, mineral exploration, and agriculture because of its high throughput, multiple channels, and high resolution.

Fourier transform imaging spectroscopy (FTIS) [7,8] acquires the data cube containing space and spectral information of the same target. Spectrum reconstruction [9,10,11,12] is the process of transformation from the interferogram to spectrogram. The remote sensing image, whose initial data are collected by the interference imaging spectrometer, is a typical application of spectrum reconstruction.

**Figure 1:**
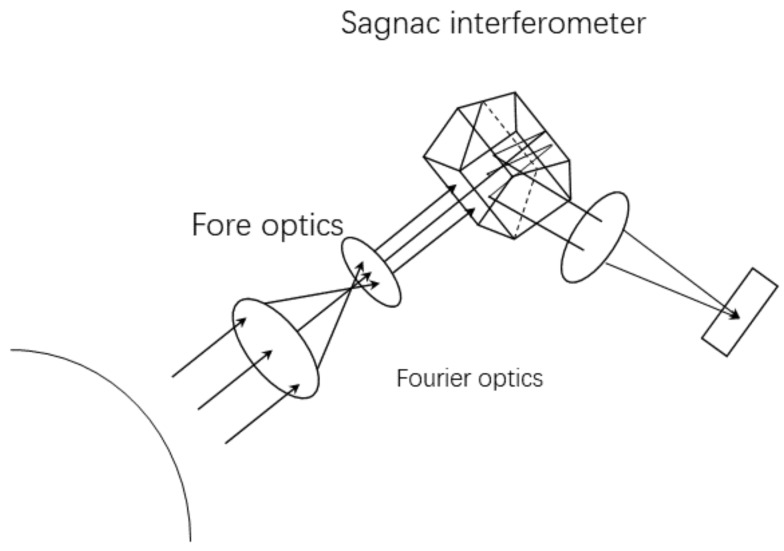
Schematic diagram of the large aperture static imaging spectrometry (LASIS) based on the Sagnac interferometer.

The equipment at the heart of spectroscopy is the interferometer. In theory and practice, usually, measures are taken to divide light from the target into two coherent light beams, which will interfere on the sensor and change the optical path difference (OPD) of the two beams in order to obtain a series of interference patterns. Figure 1 shows the principle of large aperture static imaging spectrometry (LASIS) [13].

The current Fourier transform spectrometers are able to capture large areas in increased spatial and spectral resolution, and the amount of interferograms from the sensors both in orbit and on the ground is increasing rapidly. To process the large-scale interferograms as soon as possible, we usually omit some steps so that the recovery spectrum has many side lobes and false peaks, which is not reliable. These problems should be solved in a fast processing pipeline.

In the traditional spectrum reconstruction mechanism, fast Fourier transform (FFT) is the key part. However, the spectrum reconstructed by FFT from the actual obtained interferogram extends badly and has many side lobes, which would decrease the recovery accuracy and the resolution power. In addition, the interferogram cannot be scanned to infinity as the theoretical calculation. The interferogram value, out of the scanning range, is regarded as zero, which can result in spectrum leakage. If we use apodization technology, the spectrum leakage could be solved, but the main lobe width of the power spectrum would increase. With its full width at half maximum (FWHM) becoming large, there may be spectral peaks that are hard to identify in the spectrum. Meanwhile, the spectral resolution of FFT depends on the length of the signal. In practice, especially on a satellite, it is not permitted to transmit long interference data, and there are not enough sampling points to improve the spectral resolution. When the signal is short, the spectrum is difficult to identify carefully.

To solve these problems, Jian et al. [14,15] brought modern spectrum estimation into the field of spectral reconstruction. They introduced the multiple signal classification (MUSIC) [16] algorithm and the autoregressive (AR) [17,18] model for better performance in spectral recovery. These algorithms are good at spectrum reconstruction in resolution, but are very time consuming. The MUSIC algorithm is suitable for monochromatic light spectrum reconstruction, whereas AR spectral estimation is widely applicable. Although modern spectrum estimation has many advantages in resolution and suppressing the heavy-tailed spectrum, even for a short signal, the algorithm complexity is so high that these models are restricted in practical spectrum reconstruction. Quick solutions are needed.

In recent years, programmable graphics hardware has grown significantly in terms of performance and functionality. In comparison with the traditional data processing pipeline, performing general-purpose computations on GPUs is a new means of data handling, and it is possible to achieve a significant reduction in processing time when parallelized and executed on a GPU in accelerating algorithms and batch processing. It is particularly suitable for solving problems that can be expressed as data parallel computation, that is the same program is executed on many data elements in parallel [19]. Furthermore, compared with multi-thread computation on the CPU and multi-core CPUs, the parallel computing on GPUs has thousands of threads, which are simple to create and apply. In addition, multi-thread computation on the CPU and multi-core CPUs would occupy many system resources and decrease the total performance of the system. However, on GPUs, it just needs to transfer data from the CPU to the GPU and the GPU to the CPU and would not occupy resources on the CPU. The complex computing tasks are assigned to the GPU, and this does not have an impact on the overall performance of the system. Another important advantage on the GPU is that we can process many batches of data at the same time. The more the amount of data, the higher the performance.

Compute Unified Device Architecture (CUDA) was introduced by NVIDIA, a general purpose parallel computing platform and programming model using the parallel-compute engine in NVIDIA GPUs to solve many complex computational problems in a more efficient way than on a CPU. Another advantage of CUDA is that it provides standard programming languages such as C/C++, which is familiar for programmers [20,21]. This is an effective way to deal with many interferograms to reach the goal of high resolution and fast processing in spectrum reconstruction.

This paper will focus on quick solutions of the autoregressive model for high spectral resolution with better performance in real time and the parallel processing scheme of spectrum reconstruction based on high performance parallel computing on the GPU. The main contributions of our research are as follows:

- We propose a parallel solution for high-resolution spectrum estimation. We use the parallel Burg method to solve the AR model and to obtain the model solution in a faster way. We validate the performance of our parallel algorithm by comparing it to measures on a CPU that is also used in the experiments.
- We use our parallel Burg solution for batch processing. The GPU is suitable for data parallel computation. We accelerate the application in data parallelism. Thousands of threads have been used for large-scale data processing.
- We design an asynchronous parallel processing mechanism for high-resolution spectrum reconstruction by making full use of the GPU with overlap. Within the limited threads and memory, the asynchronous parallel mechanism simultaneously executes kernels and transfers data using multiple streams. The number of streams can be set to two, three, or other values.
- Many solutions for the AR model are discussed for comparison. We use the Yule–Walker and least-squares methods for the AR model, and two recursive algorithms of the Yule–Walker equation have been employed for fast parameter estimation.

The rest of this paper is organized as follows: Section 2 discusses the related work, and Section 3 depicts a parallel algorithm and designs a parallel processing mechanism for fast parameter estimation. The experiments are arranged in Section 4. In Section 5, we give an analysis and draw conclusions.

## 2. Related Work

In this section, we will discuss the related work on the general data processing pipeline and a high-resolution model for the interferogram in the Fourier transform spectrometer. Two theoretical solutions for the model are also discussed.

### 2.1. General Data Processing

Based on the theory of Fourier transform spectroscopy [22], the spectrum could be obtained through Fourier transformation for the interferogram, listed as the following equations:

(1) $$I\left(\Delta \right)=\int_{-\infty }^{+\infty }B\left(\sigma \right)e^{j2\pi \sigma \Delta }\;d\sigma ,$$

(2) $$B\left(\sigma \right)=\int_{-\infty }^{+\infty }I\left(\Delta \right)e^{-j2\pi \sigma \Delta }\;d\Delta ,$$

where *I* is the interferogram, *B* is the spectrum, and Δ and σ mean the path difference and the wave number, respectively. The processing of the interferogram mainly includes preprocessing, apodization [23,24,25], phase correction [26,27,28], and FFT, where apodization is to eliminate the effect of the instrument linear function for power spectrum and phase correction can correct phase error. This is the most direct and simple way for spectrum reconstruction based on Equations (1) and (2) between the interferogram and spectrogram.

The traditional algorithm mainly including FFT and apodization is substantially equivalent to the BT (Blackman and Tukey) method in classical spectrum estimation. However, the classical method suffers from large estimated variance and bad resolution, which could be overcome by modern spectrum estimation, the autoregressive model for example.

### 2.2. Autoregressive Model

The autoregressive (AR) model is a linear prediction model that could be employed to predict unobserved values when given a set of known data. In theory, any random signal can be considered as a sequence produced by white noise through the model, whose parameters could be calculated by observed values so that the unobserved data are not thought to be zero. Generally, for generating random sequences, a linear difference equation is used as the system model, which is known as the *p*-order auto-regression model, as follows:

(3) $$x\left(n\right)=-\sum_{k=1}^{p}a\left(k\right)x\left(n-k\right)+\omega \left(n\right)$$

where x(n) is the random sequences, ω(n) means white noise, and a(k) is the factor. Its corresponding power spectral estimation P(ω) is:

(4) $$P\left(\omega \right)=\frac{\sigma_{\omega }^{2}}{|1+\sum_{k=1}^{p}a\left(k\right)e^{-j\omega k}{|}^{2}}$$

where $\sigma_{\omega }^{2}$ is the variance of prediction error. We can get the whole model if all a(k) and $\sigma_{\omega }$ are calculated.

For the solution of parameters, there are two theoretical solutions, the Yule–Walker equation and least-squares method, which will be discussed in the following part. The model is an excellent approximation to the stochastic process, as long as we get a good estimation of the parameters and choose an appropriate order [29,30,31,32].

#### 2.2.1. Yule–Walker Equation

From Equation (3), we can obtain the signal correlation:

(5) $$\begin{array}{cc} r_{xx}\left(m\right) & =E[x(n)x(n+m)]\\ & =E\{x\left(n\right)\left[-\sum_{k=1}^{p}a\left(k\right)x\left(n-k+m\right)+w\left(n+m\right)\right]\}\\ & =-\sum_{k=1}^{p}a\left(k\right)r_{xx}\left(m-k\right)+E\left[x\left(n\right)w\left(n+m\right)\right]. \end{array}$$

Through Equation (5), it can be inferred that x(n) is only related to w(n) and not related to w(n+m),m≥1. We can obtain the matrix representation as follows.

(6) $$\left[\begin{array}{cccc} r_{xx}\left(0\right) & r_{xx}\left(-1\right) & \cdots & r_{xx}\left(-p\right)\\ r_{xx}\left(1\right) & r_{xx}\left(0\right) & \cdots & r_{xx}\left(-p+1\right)\\ \vdots & \vdots & \ddots & \vdots \\ r_{xx}\left(p\right) & r_{xx}\left(p-1\right) & \cdots & r_{xx}\left(0\right) \end{array}\right]\left[\begin{array}{c} 1\\ a(1)\\ \vdots \\ a(p) \end{array}\right]=\left[\begin{array}{c} \sigma_{w}^{2}\\ 0\\ \vdots \\ 0 \end{array}\right]$$

This is the famous Yule–Walker equation, which is the basis of other estimation methods. The coefficients of the AR model and power spectral density can be estimated based on Equation (6). If ${\left\{r_{xx}\left(k\right)\right\}}_{k=0}^{P}$ can be computed, we can obtain:

(7) $$r_{p}+R_{p}\beta_{YW}=0,$$

where

(8) $$\beta_{YW}={\left[a\left(1\right),a\left(2\right),\cdots ,a\left(P\right)\right]}^{T},$$

(9) $$r_{p}=\left[r_{xx}\left(1\right),r_{xx}\left(2\right),\cdots ,r_{xx}\left(p\right)\right],$$

(10) $$R_{p}=\left[\begin{array}{ccc} r_{xx}\left(0\right) & \cdots & r_{xx}\left(-p+1\right)\\ \vdots & \ddots & \vdots \\ r_{xx}\left(p-1\right) & \cdots & r_{xx}\left(0\right) \end{array}\right]$$

$\beta_{YW}$ can be estimated by:

(11) $$\beta_{YW}=-R_{p}^{-1}r_{p}.$$

#### 2.2.2. Least-Squares Method

The least-squares (LS) method, widely used in data processing in engineering technology and scientific research, searches for the best matching function by minimizing the square errors. It is suitable for random and stationary signals.

Suppose there are *n* points $\left(x_{i},y_{i}\right)$ in the dataset, where $x_{i}$ is the independent variable and $y_{i}$ is the observation value, i=1,2,⋯,n. Let f(x,β) be a fitting function for the dataset, where β has *m* parameters to be estimated. Define the difference $r_{i}$ between $y_{i}$ and $f(x_{i},\beta )$ as follows,

(12) $$r_{i}=y_{i}-f\left(x_{i},\beta \right).$$

The least-squares method is to minimize the sum of $r_{i}$, that is,

(13) $$\min S=\min\frac{1}{2}\sum_{i=1}^{n}r_{i}^{2}.$$

Here, we give the estimation result for β in a matrix form directly (see [33] for more details).

(14) $$\hat{\beta }={\left(X^{T}X\right)}^{-1}X^{T}y.$$

In the AR model, Equation (3) could be written in the following formula:

(15) y=Xβ+w,

where $y={\left[x_{p+1},x_{p+2},\cdots ,x_{N}\right]}^{T}$, β=[a(1),a(2),⋯,a(p)], w=[w(p+1),w(p+2),⋯,w(N)] and:

(16) $$X=\left[\begin{array}{cccc} x_{p} & x_{p-1} & \cdots & x_{1}\\ x_{p+1} & x_{p} & \cdots & x_{2}\\ \vdots & \vdots & \ddots & \vdots \\ x_{N-1} & x_{N-2} & \cdots & x_{N-p} \end{array}\right].$$

In fact, we would obtain the parameters’ estimation of the AR model directly through Equation (14). In some cases, the matrix decomposition of *X* is also a nice solution for β. When *X* is a full-rank matrix with $X\in R^{m\times n}$, that is, if m≥n, rank(X)=n. *X* can be decomposed by QR factorization,

(17) X=QR,

where *Q* is an orthogonal matrix, meaning $Q^{T}Q=I$, and *R* is an upper triangular matrix [33]. It can be rewritten as:

(18) $$\hat{\beta }=R^{-1}Q^{T}y,$$

which means the least-squares method is converted into QR decomposition and matrix inversion. The parallel QR decomposition and matrix inversion [12] should be taken into account in parallel application, which we will not discuss in detail.

## 3. Review of the AR Model

In this section, we mainly discuss the parallel recursive algorithm and mechanism of high resolution and fast processing of spectral reconstruction based on high-performance parallel computing. Before that, the recursive algorithms for the Yule–Walker equation are discussed first.

### 3.1. The Recursive Algorithms for the Yule–Walker Equation

It is difficult to estimate all coefficients of the Yule–Walker equation and least-squares method because they need matrix inversion, so that it would add a heavy computational burden when the order *P* is large. Two famous recursive algorithms, Levinson–Durbin and Burg estimation, have been employed in solving the Yule–Walker equation.

Levinson et al. [34,35] proposed a high-performance recursive algorithm by successively estimating $\left\{a\left(1,1\right),\sigma_{1}^{2}\right\}$, $\left\{a\left(2,1\right),a\left(2,2\right),\sigma_{2}^{2}\right\}$, ⋯, $\left\{a\left(p,1\right),a\left(p,2\right),\cdots ,\sigma_{P}^{2}\right\}$. The result with *p* order is the required solution of Equation (6). The recursive relation is listed as follows.

(19) $$\left\{\begin{array}{c} a\left(p,p\right)=-\frac{1}{\sigma_{p-1}^{2}}\left[r_{xx}\left(p\right)+\sum_{k=1}^{p-1}a\left(p-1,k\right)r_{xx}\left(p-k\right)\right]\\ a(p,k)=a(p-1,k)+a(p,p)a(p-1,p-k),k=1,2,\cdots ,p-1\\ \sigma_{p}^{2}=\left[1-a{\left(p,p\right)}^{2}\right]\sigma_{p-1}^{2},\sigma_{0}^{2}=r_{xx}\left(0\right) \end{array}\right.$$

The Levinson–Durbin (L-D) recursive algorithm is used to solve the coefficients of the AR model. Although it can simplify computation, we still need to know the autocorrelation sequence $r_{xx}\left(k\right)$, which could only be calculated from finite time series. When the data length *N* is small, the computation error will become large.

Under the constraint of the L-D recursive relation, Burg et al. proposed a recursive algorithm to minimize the sum of a priori and a posteriori prediction error energy to evaluate all coefficients.

According to the linear prediction theory, $\hat{x}$ can be indicated by the weighted sum of the previous value, that is,

(20) $$\hat{x}\left(n\right)=-\sum_{k=1}^{p}a\left(p,k\right)x\left(n-k\right).$$

The a priori prediction error can be written as:

(21) $$e\left(p,n\right)=x\left(n\right)-\hat{x}\left(n\right)=x\left(n\right)+\sum_{k=1}^{p}a\left(p,k\right)x\left(n-k\right).$$

In Equation (19), let the reflection coefficient k(p)=a(p,p), and define the a posteriori prediction error:

(22) $$b\left(p,n\right)=x\left(n-p\right)+\sum_{k=1}^{p}a\left(p,k\right)x\left(n-p+k\right),$$

the recursive formula is

(23) e(p,n)=e(p−1,n)+k(p)b(p−1,n−1),

similarly,

(24) b(p,n)=b(p−1,n−1)+k(p)e(p−1,n).

For better estimation of k(p), the Burg algorithm is based on the principle of minimum mean squared error, i.e., $\min[e^{2}+b^{2}].$ k(p) is evaluated by:

(25) $$k\left(p\right)=-\frac{2E[e(p-1,n)b(p-1,n-1)]}{E[e^{2}\left(p-1,n\right)+b^{2}\left(p-1,n-1\right)]}.$$

For a stationary random process,

(26) $$\hat{k}\left(p\right)=-\frac{2\sum_{n=p}^{N-1}e\left(p-1,n\right)b\left(p-1,n-1\right)}{\sum_{n=p}^{N-1}\left[e^{2}\left(p-1,n\right)+b^{2}\left(p-1,n-1\right)\right]}$$

and:

(27) e(0,n)=b(0,n)=x(n),

(28) $${\hat{\sigma }}_{0}^{2}={\hat{r}}_{xx}\left(0\right)=\frac{1}{N}\sum_{n=0}^{N-1}x^{2}\left(n\right).$$

All parameters could be calculated from the following iterative relation.

(29) $$\left\{\begin{array}{c} a\left(p,k\right)=a\left(p-1,k\right)+\hat{k}\left(p\right)a\left(p-1,p-k\right)\\ \sigma_{p}^{2}=\left[1-{\hat{k}}^{2}\left(p\right)\right]\sigma_{p-1}^{2} \end{array}\right.$$

### 3.2. Parallel Burg Recursive Algorithm

The AR model for spectral estimation would provide a higher resolution; meanwhile, it needs more time to compute the parameters due to its high algorithm complexity, especially for large data and batch processing. In order to process huge data using the high-resolution method for spectrum reconstruction in a more effective way, we parallelized the Burg method for the extension of its applicability. The parallel Burg (P-Burg) algorithm mainly contains two parts: initialization and update.

Both initialization and update involve the dot product on the GPU. Given two vectors *G* and *H* with the same length *N*, we calculate the dot product through:

(30) $$V=G\cdot H=\sum_{i=0}^{N-1}g_{i}h_{i}.$$

**Figure 2:**
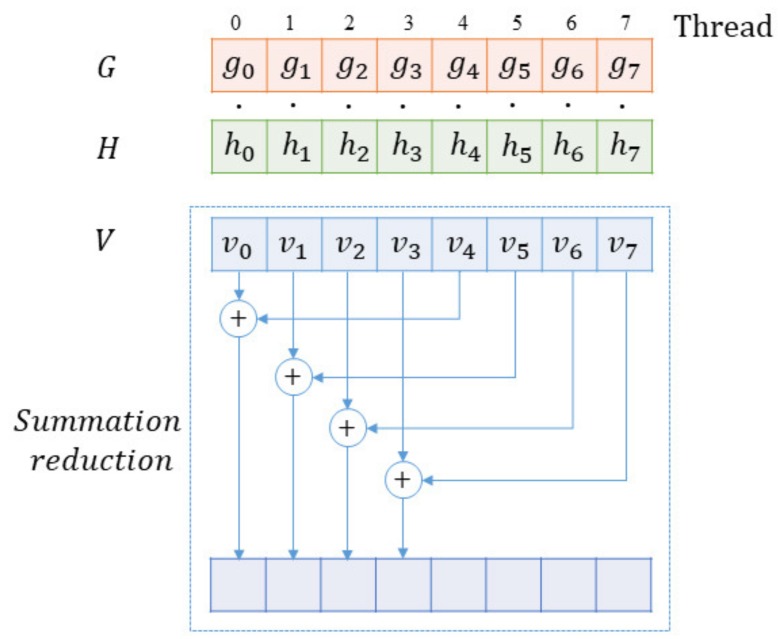
Parallel dot product for two vectors and a step of summation reduction on the GPU.

For threads on a GPU, some of them are used for summation reduction besides completing the computing task of multiplying the corresponding elements. In summation reduction, since each thread combines two entries into one, we complete this step with half as many entries as we started. In the next step, we do the same thing on the remaining half. Thus, it can be seen that there would be logN steps with *N* (*N* is a power of two). After these steps of summation reduction, the final result would be recorded in $v_{0}$, illustrated by Figure 2. Specifically, shared memory could be used for faster addressing and better performance. When the summation reduction begins, we declare a buffer of shared memory in a block, which will be used to store each thread’s running sum. When all the threads finish the computation task, the buffer is filled up. Then, we can sum the values in the buffer to accomplish the reduction, as Figure 2 shows. For example, if we use 256 threads per block, 256 values from the shared memory would be used to form 128 values through summing every two values into one. In the second iteration, 128 values would be summed into 64 values. Until the last iteration, the reduction is completed. It will take eight iterations to reduce the 256 values into a single sum.

Specifically, slightly different from the representation in Section 2, in this section, e,b,a,k are considered as row vectors, and $a_{m}\left(j\right)$ means the $j^{\mathrm{th}}$ element of the vector *a* in the $m^{\mathrm{th}}$ iteration. Furthermore, the index of a vector starts from zero.

#### 3.2.1. Initialization

We usually initialize the parameters by:

(31) $$e_{0}\left(tid\right)=b_{0}\left(tid\right)=x\left(tid\right),$$

(32) $$\sigma_{0}^{2}=\frac{1}{N}x\cdot x,$$

where tid means the index of the current thread and tid=0,1,⋯,N−1 means that we arrange *N* threads to compute the dot product. The first element of *a*, that is a(0), is set to one.

#### 3.2.2. Update

After initialization, the parameters will be updated iteratively. In one iteration, the reflection coefficient *k* is firstly updated by:

(33) $$\hat{k}\left(m\right)=-\frac{2e_{m-1}\cdot b_{m-1}}{e_{m-1}\cdot e_{m-1}+b_{m-1}\cdot b_{m-1}}.$$

**Figure 3:**
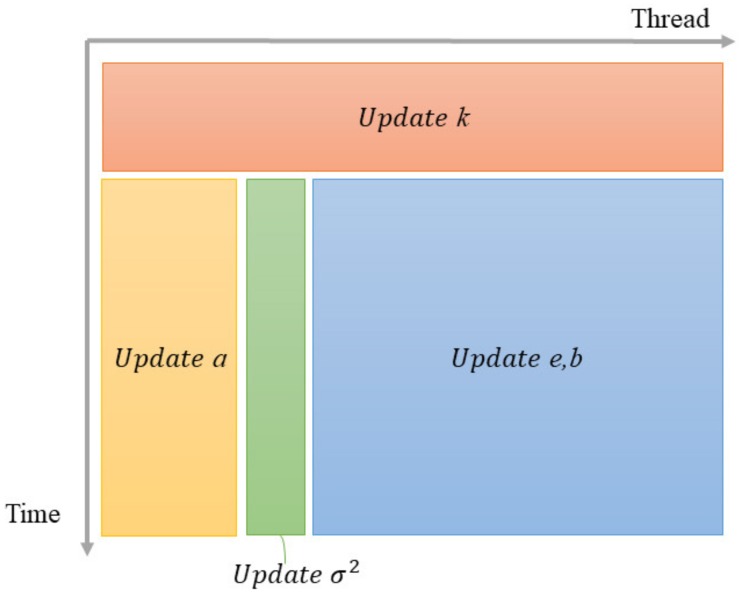
The flowchart of the parallel update in one iteration.

Then, a(p), $e_{p}$, $b_{p}$ and $\sigma_{p}^{2}$ are updated at the same time through:

(34) $$e_{m}\left(tid\right)=e_{m-1}\left(tid+1\right)+\hat{k}\left(m\right)b_{m-1}\left(tid+1\right),$$

(35) $$b_{m}\left(tid\right)=b_{m-1}\left(tid\right)+\hat{k}\left(m\right)e_{m-1}\left(tid\right),$$

(36) $$a_{m}\left(j\right)=a_{m-1}\left(j\right)+\hat{k}\left(m\right)a_{m-1}\left(m-j-1\right),$$

(37) $$\sigma_{m}^{2}=\left[1-\hat{k}{\left(p\right)}^{2}\right]\sigma_{m-1}^{2},$$

where j=0,1,⋯,m, *m* indicates the iteration number, m=1,2,⋯,p, and tid is the index of the current thread, tid=0,1,⋯,N−p. Figure 3 shows the operation flow of the P-Burg method in one iteration on a GPU.

When we update *a* based on Equation (36), it should be updated for all $a_{p}\left(j\right)$ at the same time, and we can set *m* threads to update the *m* parameters, listed as follows,

(38) $$a_{m}\left(tid\right)=a_{m-1}\left(tid\right)+\hat{k}\left(p\right)a_{m-1}\left(m-1-tid\right),$$

where tid=0,1,⋯,m−1.

It should be noted that model order *p* determines the number of iterations, and we do not take the cuBLAS library (an implementation of basic linear algebra subprograms on top of CUDA runtime) into account. The reasons are listed as follows. We do not have enough sampling points from the interferogram in the experiments for a long dot reduction. At this order of magnitude, our routine for dot product computation has almost the same performance as the one included in the cuBLAS library. When enough data are available, the performance is much better with the dot product in the cuBLAS library. Another important reason is that in Equation (33), we need three different dot products, and there are operations between the three values. If we used cuBLAS, we would still need to create a new kernel for the only three values, which would be hardly efficient on the GPU. In our routine, we can get the result of the three dot products at the same time and calculate them in the same kernel. This is more flexible in solving our problems.

### 3.3. Parallel Processing Mechanism

Generally speaking, parallel tasks are completed by the collaboration of the CPU and GPU, where the CPU is responsible for flow control and data cache, while the GPU is dedicated to solving problems that can be represented as data parallel computing, that is the same programs are executed in a parallel way on many data elements. However, resources on the GPU are limited, especially for batch processing. With the limited memory and threads, we take advantage of the characteristics of the GPU with overlap to design an asynchronous parallel processing mechanism for spectrum reconstruction when it comes to batch processing.

Stream plays an important role in accelerating applications. It represents a GPU operation queue where the operations will be executed in a specified order. We can add some operations in the stream, such as kernel function, memory copy, etc. The order in which these operations are added to the stream is also their execution order. Each stream can be thought of as a task on the GPU, and these tasks can be executed in parallel.

**Figure 4:**
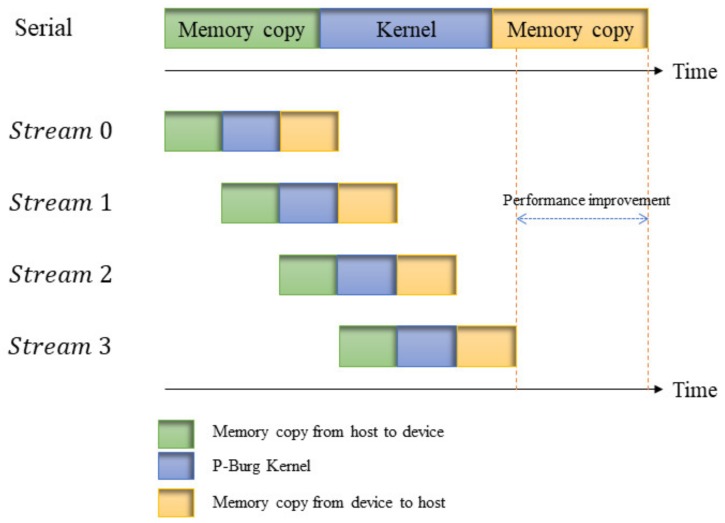
Example of the parallel processing mechanism based on overlap.

Figure 4 shows an example of the parallel processing mechanism based on four streams. In the figure, the kernel in the serial pipeline comprises four small P-Burg kernels, which are implemented one after the other. In the parallel pipeline, the four P-Burg kernels are arranged in the four streams, respectively, which could be executed in an asynchronous way. In synchronization mode, we successively execute the memory copy from the host (CPU) to the device (GPU) and the kernel and memory copy from the device to the host. However, in the asynchronous parallel processing mechanism, tasks are divided into several streams. When Stream 0 executes a kernel, data transfer from the host to the device in Stream 1, and when Stream 1 executes the kernel, memory copies from the device to the host and from the host to the device are executed in Stream 0 and Stream 2, respectively. Thus, the performance improves because the GPU that support overlap can execute the memory copy between the device and the host while executing our P-Burg kernel function.

### 3.4. Comparison with FFT

The interferogram cannot be obtained ranging from negative infinity to positive infinity in Equations (1) and (2). The optical path difference Δ in practical application satisfies the following relation,

(39) −L≤Δ≤+L,

where *L* is the maximum optical path difference. In Fourier transform spectroscopy, the resolution of FFT is the reciprocal of the data length *N*. This value, which falls out of the scanning range, is considered as zero, which is equivalent to multiplication of the signal and a rectangular window. In the spectrum, a rectangular window has many side lobes, leading to spectrum leakage. We could choose an appropriate function such as the Hamming window, the triangular window, the Happ–Genzel window and Bessel window in apodization technology. Through apodization with a window function, the FHWM increases. Meanwhile, the AR model is to estimate the interferogram rational value out of the scanning range instead of zeros according to its principle. From this point, this means more data are used to reconstruct the spectrum, and then, the resolving power is improved. In addition, the model solved by P-Burg has fewer side lobes, and its FWHM is smaller than the one of FFT, which will be verified in Section 4.

In terms of the operation time of the algorithms, FFT has the best performance because of its low complexity (NlogN). Even using our parallel Burg recursive solution, the model still needs much more time to estimate the parameters.

## 4. Experiments

Our experiments were implemented using C/C++ and CUDA C under Ubuntu 16.04. The computer configuration was as follows: Inter Xeon CPU E5-2609, 16 GB RAM, and Quadro K620 graphics card. Table 1 lists more information about the K620 card.

**Figure 5:**
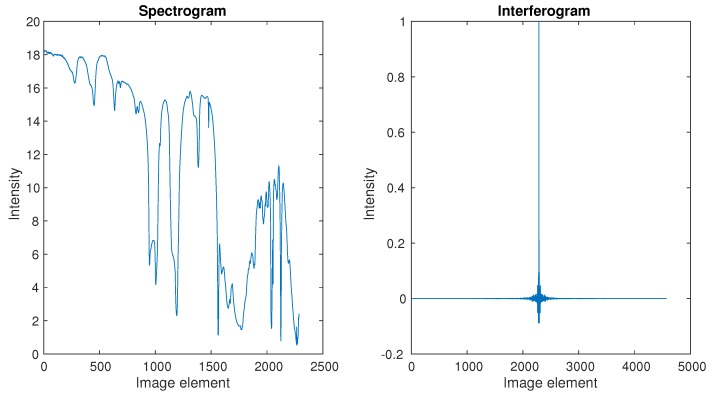
Spectrogram and its corresponding interferogram from JPL Lab.

**Figure 6:**
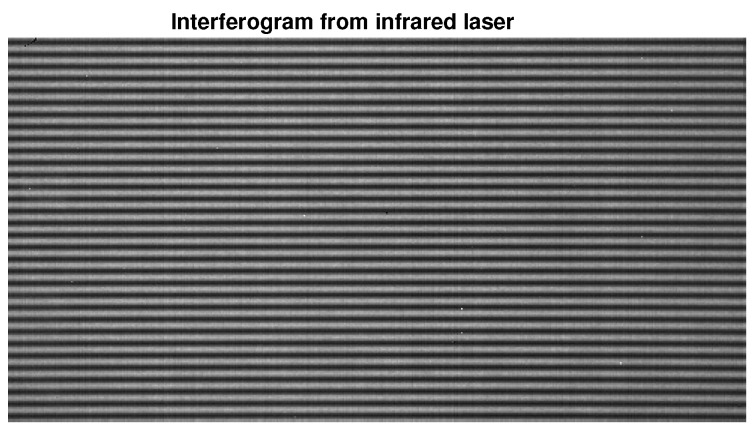
A frame of the laser interferogram whose wavelength is 1550 nm.

**Figure 7:**
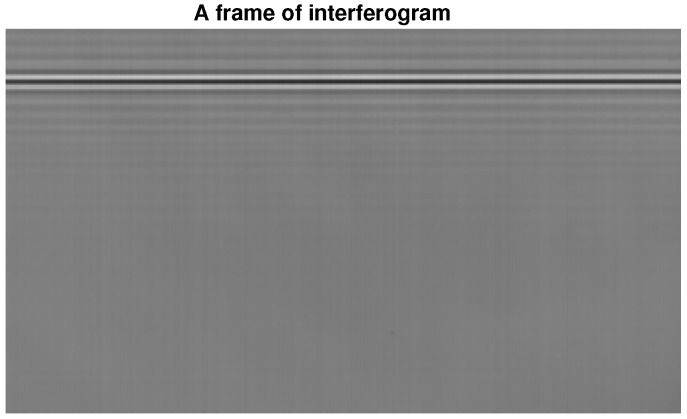
A frame of the white light interferogram from LASIS.

The famous JPL Lab provides the spectrum for our simulations, and it could be seen as an ideal spectrum, shown by Figure 5. Its corresponding interferogram was transformed, which could be seen as an ideal interferogram. Our actual laser and white light interferograms were provided by the LASIS interferometer in our practical application, as shown in Figure 6 and Figure 7 respectively.

### 4.1. Reconstruction Result

**Figure 8:**
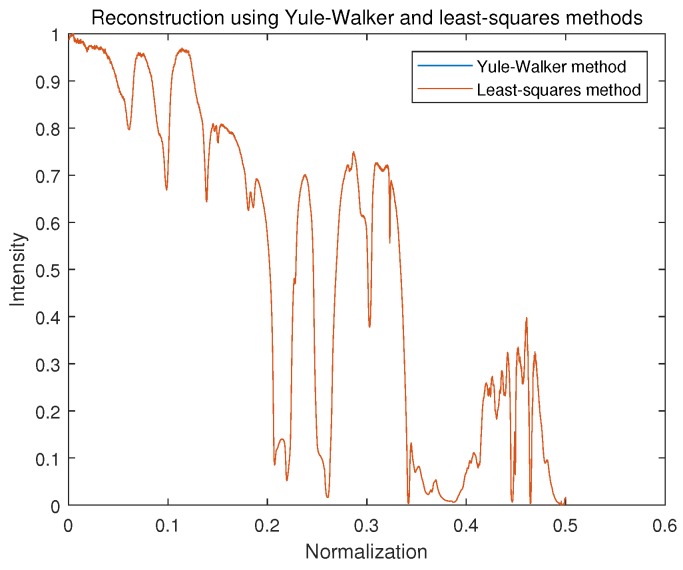
Spectrum reconstruction using the Yule–Walker method and least-squares method.

We compared the Yule–Walker and LS methods to solve AR model using the ideal interferogram from Figure 5. The result of spectrum reconstruction is illustrated in Figure 8. We can see that both the Yule–Walker method and LS method provided a high level consistency, and there was little difference between them. It should be noticed that the construction spectrum using the AR model was a power spectrum, and the general tendency of the original spectrum was recovered by the two methods.

**Figure 9:**
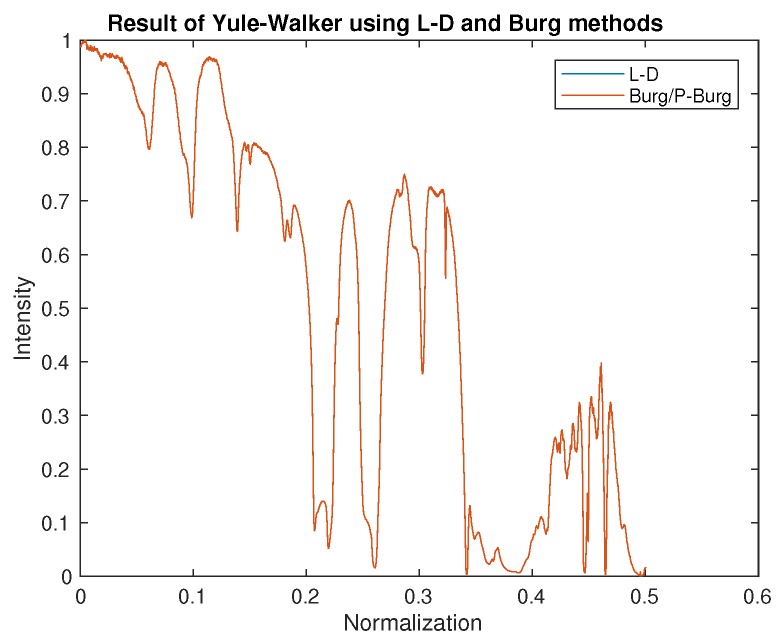
The result of the spectrum reconstruction using the Yule–Walker recursive algorithm, Levinson–Durbin (L-D), and Burg/parallel Burg (P-Burg).

Figure 9 shows the result of two Yule–Walker recursive algorithms, the Levinson–Durbin algorithm, and the Burg (P-Burg) algorithm. It can be seen that these two methods could estimate the parameters of AR well, and because matrix inversion was not involved during the solving process, both were much faster than Yule–Walker and LS. The reconstruction results from Figure 8 and Figure 9 are almost the same.

### 4.2. FFT and P-Burg

**Figure 10:**
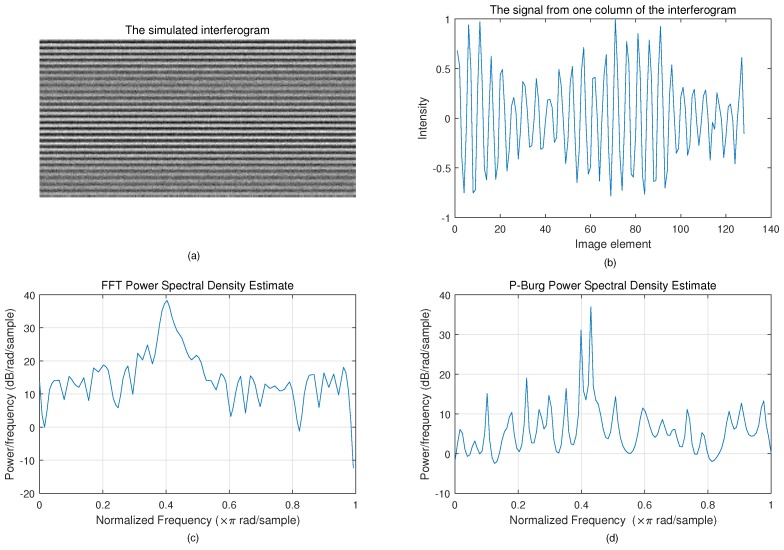
Spectrum reconstruction using FFT and P-Burg for a signal from the simulated interferogram. (**a**) The simulated interferogram. (**b**) A signal from one column of the interferogram. It consists of two different-frequency sine signals, whose frequency is very close, and white noise. The length of the signal is 128. (**c**) The power spectral density using FFT. (**d**) The power spectral density using P-Burg.

**Figure 11:**
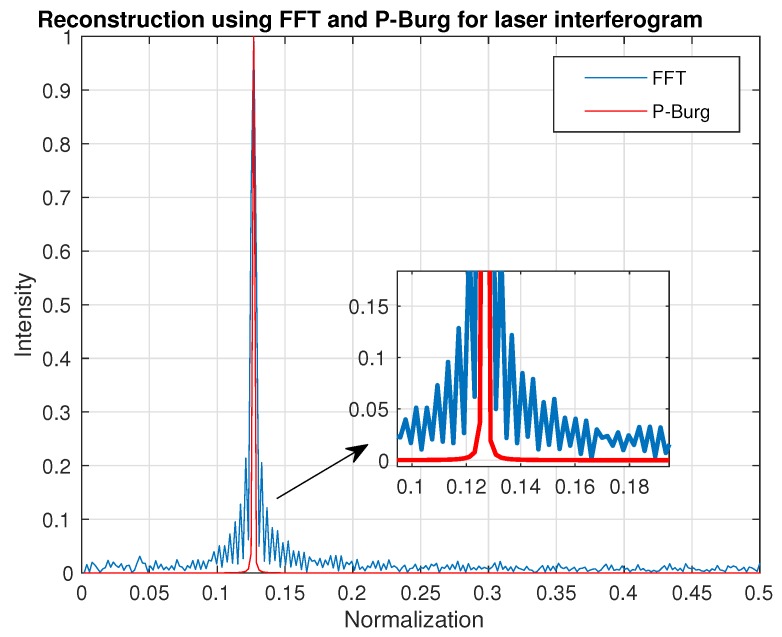
Spectrum reconstruction using FFT and P-Burg for the laser interferogram.

Figure 10 shows the result of spectrum reconstruction using FFT and P-Burg for a simulated interferogram. We can see that the P-Burg method can identify the two spectrum peaks (Figure 10d), while there is only one peak in the power spectral density using FFT (Figure 10c). FFT can only identify them when the length of the signal increases. Figure 11 shows the reconstruction result of the laser interferogram (Figure 6). Obviously, the spectrum of FFT has more side lobes and larger FWHM than the one reconstructed by P-Burg. It can be verified that P-Burg has better performance in power resolution for spectrum reconstruction from the two figures.

Table 2 shows the runtime of FFT and P-Burg for our laser interferogram where the length N=256 and the model order *p* is set to five. The FFT has the best performance in runtime. Although FFT is much faster to perform than P-Burg by one order of magnitude, P-Burg tries to keep the high resolution in spectrum reconstruction while meeting the real-time requirements. An interesting thing is that the parallel FFT on the GPU runs slower than the FFT on the CPU. This is because when *N* is small, the time consumed by communication between threads can be comparable to the computation time. This leads to a decline in parallel FFT performance.

### 4.3. Performance of Burg and Parallel Burg

**Figure 12:**
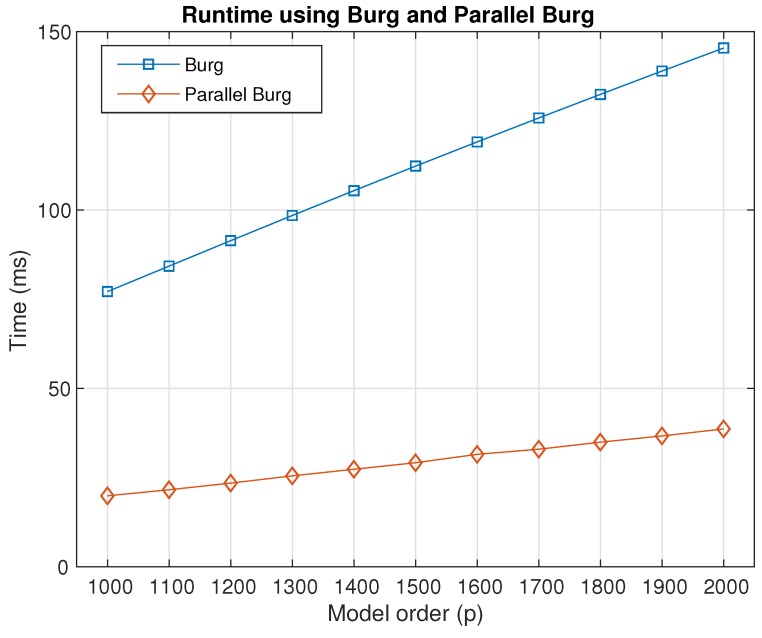
Runtime between the Burg algorithm and the parallel Burg algorithm with model order.

Figure 12 shows the runtime cost by Burg and parallel Burg with the increase of the model order *p*. The length *N* is 4573. For every order *p*, we ran the algorithms a thousand times and measured the performance by the average of the total time for the two methods, respectively. The runtime cost by the Burg and parallel Burg methods could increase as the model order becomes larger. In addition, the time cost by parallel Burg was much lower than that cost by Burg, and it can be concluded that the parallel Burg algorithm had a higher performance in algorithm efficiency compared with the traditional Burg method.

Table 3 shows the ratio and improvement between Burg and parallel Burg. The ratio, improvement, and MSE were calculated by the following equation:

(40) $$ratio=\frac{Time_{Burg}}{Time_{P-Burg}},$$

(41) $$improvement=\frac{Time_{Burg}-Time_{P-Burg}}{Time_{Burg}}.$$

(42) $$MSE\left(a\right)=\frac{1}{p+1}\sum_{i=0}^{p}{\left[a{\left(i\right)}_{P-Burg}-a{\left(i\right)}_{Burg}\right]}^{2}.$$

In the table, the time consumption of Burg is about 3.8-times that of P-Burg with the model order ranging from 1000–2000, that is the efficiency of P-Burg was 3.8-times as high as that of Burg. The improvement was about 74%, which means the performance increased by 74%. In terms of the estimation accuracy, the estimated parameters of P-Burg was almost the same as the serial result. The difference was only the system calculation error. In fact, the other solutions of AR model had consistent parameter estimation when the length *N* and the order *p* were the same. This is, in total, a satisfactory result.

Despite the high performance of P-Burg, it should not be forgotten that the parallel Burg method was executed on the GPU and occupied many GPU resources, including memory and threads. That is the advantage of the GPU in accelerating applications.

### 4.4. Batch Processing for Large Data Using P-Burg

The GPU is a powerful tool for dealing with large amounts of data, and it plays a very important role in batch processing or data parallelism. That means if we compute an original signal with length *N*, then we have *T* groups of similar signals, and these groups of signals are operated the same as the original one. In our experiment and simulation, we set N=4573, and *T* ranged from 10–100. Compared with the CPU, the GPU had better performance for computing, proven by Table 4, and the efficiency ratio was about 5–10.

In the table, we can see that the time required for processing on the CPU linearly increased as the value of *T* were from 10–100, while the runtime cost on the GPU also increased gradually. That may be different from the ideal situation since more and more threads and memory are configured to complete the task, and it should not increase with *T*. The reason is that thousands of threads need to collaborate with each other, and these threads do not work at the same time, though they are scheduled simultaneously. Meanwhile, the more data, the more the address operations, which would result in much time delay. Furthermore, large data transmission takes much time including data copy from the CPU to the GPU and from the GPU to the CPU.

It should be recognized that we have enough resources for each group of data.

Table 5 shows some parameters from profiling information about memory transfers and GPU utilization (the number of groups was set to 100), where HtoD means memory copy from the host to device and DtoH means memory copy from the device to host. The computation occupies most of the time. In total, the parallel pipeline we designed performed well. However, the memory copies did not fully use the available host to device bandwidth because the throughput of HtoD was only 6.305 GB/s. It could be better optimized for larger throughput.

### 4.5. Parallel Mechanism with Overlap

With the resource limitation on the GPU, it should be realized that more workload will be arranged on the threads for batch processing. In other words, if we use *n* threads working on one group of data, with the limitation of threads, we could only use *n* threads for *T* groups, that is each thread will be responsible for more calculations.

**Figure 13:**
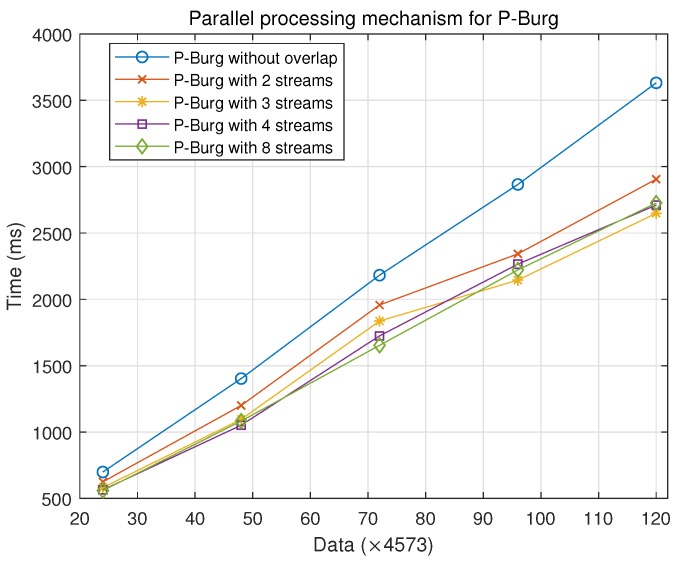
Runtime of the parallel processing mechanism for P-Burg with the limitation of threads and memory.

Figure 13 describes the performance of different parallel processing mechanisms. P-Burg without overlap means that it maintains the data copy from the host to the device, the kernel calculation and data copy from the device to the host, which is the specific execution order for many groups on the same threads without multiple streams. We take advantage of overlap and use multiple streams for P-Burg with *m* streams, m=2,3,4,8.

From Figure 13, under different data groups, the performance of the parallel processing mechanism based on multi-stream was higher than that of common parallel mode. Table 6 shows the performance improvement using different numbers of streams, compared with the serial mechanism (one stream). The multiple streams on the GPU could improve the processing efficiency by about 15%–25% for many batches of data. Compared with P-Burg with different numbers of streams, the performance is almost the same, except the two streams. Strictly speaking, the parallel mechanism of P-Burg with three streams may have higher performance with the increasing amount of data.

### 4.6. Practical Application

**Figure 14:**
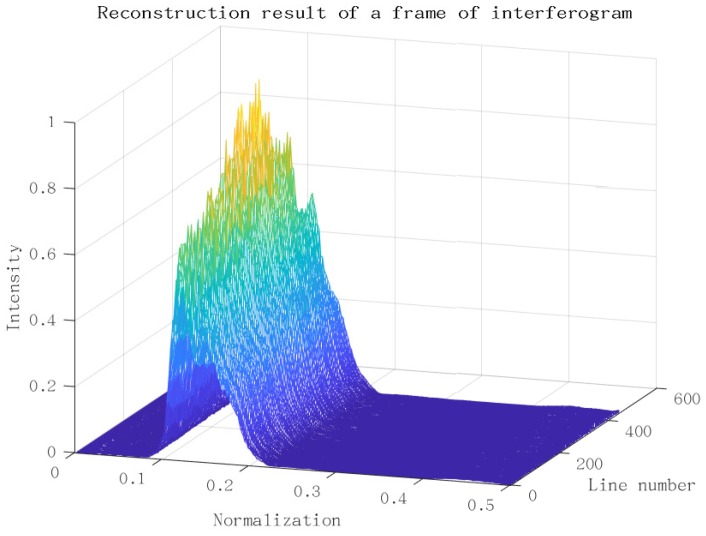
Processing result of a frame of the interferogram.

Figure 14 shows the reconstruction of a frame of the interferogram from Figure 7 using the P-Burg method. Before that, we removed the trend item of interferogram and corrected the phase error. The figure is a 3D depiction arranging all the transform results of each column in Figure 7 together. We get the result of a frame within only about 50 ms.

## 5. Conclusions

In the field of spectrum reconstruction, both the resolution and time spent in data processing are of vital importance. However, high resolution spectral reconstruction has high algorithm complexity. It needs more time to complete the task.

In this paper, a parallel high resolution algorithm in spectrum reconstruction dealing with the interferogram has been explored for fast operation. To make full use of the resources of the GPU, we design spectrum reconstruction with the P-Burg method based on the overlap. In addition, the Yule–Walker and least-squares methods solved for the AR model have been discussed for comparison. The analytical and experimental results are in good agreement. It is important to note that the AR model is sensitive to model order, though it has higher resolution in spectrum analysis. Therefore, it is necessary to select an appropriate model order using model selection criteria such as AIC, BIC, etc.

## Figures and Tables

**Table 1:** Details of Quadro K620.

CUDA driver version	9.0
CUDA capability	5.0
Global memory	1992 MB
GPU Max clock rate	1124 MHz
Shared memory per block	49,152 B
Max dimension of a block	(1024 1024 64)
Max dimension of a grid	(2,147,483,647 65,535 65,535)

**Table 2:** Runtime of P-Burg and FFT with apodization.

Method	Runtime (ms)
P-Burg	0.116
FFT with apodization	0.011
Parallel-FFT with apodization	0.020

**Table 3:** Performance comparison between Burg and parallel Burg.

*p*	1000	1100	1200	1300	1400	1500	1600	1700	1800	1900	2000
ratio	3.88	3.90	3.90	3.86	3.86	3.85	3.78	3.82	3.79	3.79	3.76
improvement (%)	74.2	74.4	74.3	74.1	74.1	74.0	73.5	73.8	73.6	73.6	73.4
MSE ($\times 10^{-32}$)	8.502	7.545	7.331	6.764	7.215	7.403	5.498	6.289	4.890	5.263	4.804

**Table 4:** Batch processing on CPU and GPU.

Groups	10	20	40	60	80	100
**CPU (s)**	1.119	2.244	4.490	6.754	8.959	11.250
**GPU (s)**	0.135	0.290	0.858	1.311	1.758	2.214

**Table 5:** Some details of profiling about GPU utilization. HtoD, host to device; DtoH, device to host.

HtoD: total bytes	12.174 MB
HtoD: throughput	6.305 GB/s
DtoH: total bytes	2.402 MB
DtoH: throughput	6.427 GB/s
Compute utilization	79.9%
Dot kernel proportion	43.6%
Sum kernel proportion	1.2%
Update kernel proportion	55.2%

**Table 6:** Performance improvement in different numbers of streams (%).

Groups	24	48	72	96	120
**2 streams**	10.59	14.43	10.29	18.25	19.99
**3 streams**	16.86	21.87	15.84	25.15	27.07
**4 streams**	19.28	25.05	20.97	20.95	25.37
**8 streams**	20.04	22.81	24.23	22.48	24.93
